# Homœopathic Medical Education

**Published:** 1887-04

**Authors:** 


### HOMŒOPATHIC MEDICAL EDUCATION.

S. L.

With pleasure, and with grief we read in the February number of THE HOMŒOPATHIC PHYSICIAN the jeremiad of Dr. Wells, of Brooklyn, on medical education, and we agree with him that there is too much *talk*, and very little done to remedy the defects. It would be dreadful if there were no means to remedy this evil; but mere talk and grumbling will not do it, nor has it ever done any good. Let our healers, who will not abate one iota from the pages of the *Organon*, put their shoulders to the wheel and start a new college, where nothing but the strictest Homœopathy shall be taught. The trial has never been made; let it be made, and success will crown their efforts. For the study of the usual branches taught in medical colleges the students might matriculate in any so-called regular college; there will be time enough left in the twenty-four hours to listen for a couple of hours to the teachings of Hahnemann and to undo the false precepts of the three P's and others. It may be a sacrifice of time and of money for the teachers to devote themselves, even at unreasonable hours, to the spread of the gospel of Hahnemann; but there are men who will not shrink from the sacrifice, if only the trial were made. We have nothing to do with the question: Will it hurt the already existing homœopathic colleges? We only believe in the survival of the best, and if there is none to be trusted, let us have one by all means.

Mere talk and grumbling will not help the cause. I, even, have the utmost confidence that there are enough physicians, members of the legion of honor, to fill up every chair taught in any other college, and with satisfaction and benefit to the students. Let our old and highly esteemed friend of Brooklyn, let Lippe, in Philadelphia, and others take the initiative; there are a few even in New York who will do their duty. By concerted action much can be achieved, which must fail by letting the hands rest in the lap and grumble.

